# Supplementary material for: Evolutionary history determines how plant productivity responds to phylogenetic diversity and species richness
Source: PeerJ. 2014 Mar 13;2:e288. doi: 10.7717/peerj.288 (PMC3961147; doi:10.7717/peerj.288)
Supplement: Appendix S1 — The following table shows the percent difference between the expected value for (1) our actual, randomly constructed mixtures and (2) hypothetical mixtures in which all species were represented equally at each level of diversity. Positive values show that the random selection picked species with above-average monoculture values for a given trait, while negative values show the opposite. The largest deviation was 2.31% and all but one of the values are less than 1.00%. [file peerj-02-288-s002.docx]

**Appendix 1.** The following table shows the percent difference between the expected value for 1) our actual, randomly constructed mixtures and 2) hypothetical mixtures in which all species were represented equally at each level of diversity. Positive values show that the random selection picked species with above-average monoculture values for a given trait, while negative values show the opposite. The largest deviation was 2.31% and all but one of the values are less than 1.00%.

|  | 3-Species | 6-Species | Within-Subgenus | Between-Subgenus |
| --- | --- | --- | --- | --- |
| Height | 0.48% | 0.94% | 0.57% | 0.97% |
| Stem Diameter | 0.21% | 2.03% | 0.90% | 0.10% |
| Survival | -0.02% | 0.36% | 0.50% | -2.31% |
